# Severity of self-reported diseases and symptoms in Denmark

**DOI:** 10.1186/1478-7954-4-3

**Published:** 2006-04-18

**Authors:** Kim Moesgaard Iburg, Niels Kristian Rasmussen, Kirsten Avlund

**Affiliations:** 1 Division of Information, Evidence and Communication, World Health Organization, Regional Office for Europe, Scherfigsvej 8, DK-2100 Copenhagen, Denmark; 2 National Institute of Public Health, Copenhagen, Denmark; 3 Department of Social Medicine, Institute of Public Health, University of Copenhagen, Denmark

## Abstract

**Objective:**

To estimate and rank the relative severity of self-reported diseases and symptoms in Denmark.

**Method:**

The 1994 Danish Health and Morbidity Survey collected data from 5,472 Danes older than 16 years of age. Interviews (response frequency: 79%) gave information on diseases and symptoms; a self-administered SF-36 questionnaire (response frequency: 64%) provided information on health-related quality of life. The severity of diseases and symptoms was represented by the health-related quality of life scores that individuals suffering from particular diseases and symptoms obtained on the single dimensions of the SF-36 and on a combined sum of all dimensions. We applied logistic regression to control for the influence of sex, age and socio-economic status on the SF-36 score. We also analysed the interaction between socio-economic status and diseases on the SF-36 score.

**Results:**

Females, more frequently than males, reported on all symptoms and all disease groups except injuries. People with relatively low levels of education reported most diseases, especially musculoskeletal and cardiovascular diseases, more frequently than people with higher education. Age-adjusted mean SF-36 scores for all dimensions combined showed that the symptoms of melancholy/depression and breathing difficulties, psychiatric disorders and respiratory diseases scored lowest (i.e. were most often associated with worse health). Females had lower SF-36 combined scores (worse health) than males on all symptoms. We found interaction between socio-economic status and respiratory diseases and musculoskeletal diseases on the SF-36 score. SF-36 scores also indicated significantly worse health among Danes with low education and income levels compared to those with higher education and income.

**Conclusion:**

In 1994 the Danes most frequently reported musculoskeletal symptoms and diseases. Psychiatric disorders and respiratory diseases were identified as the most severe reported diseases. Due to the interaction between socio-economic status and some diseases, severity estimates should be interpreted with caution or stratified by socio-economic groups.

## Introduction

For the purposes of prioritizing and planning of health care resources, it is important to obtain information on the degree to which each single disease constitutes a burden. The societal burden of a disease is a combination of its prevalence, duration and severity. The purpose of this study is to estimate and rank severities for the most prevalent self-reported diseases and symptoms in Denmark based on individual self-administrated SF-36 health-related quality of life profiles.

Based on a review of more than a thousand studies worldwide using the SF-36 questionnaire, Ware [1] concluded that the SF-36 is useful for descriptive purposes such as documenting differences between diseased patients and ones with good health levels, and for estimating the relative burden of different medical conditions. In this study we will estimate the relative severity of diseases and symptoms based on the scores that individuals suffering from these self-reported diseases and symptoms obtained on the single dimensions of the SF-36 and on all dimensions combined. We will examine how the SF-36 scores vary by age, sex, socio-economic status (SES), and will rank symptoms and disease groups according to their relative severity.

## Data and methods

The data used in this study are from the 1994 Danish Health and Morbidity Survey of the National Institute of Public Health in Denmark [2]. The total sample of 6,787 adults older than 16 years of age comprised a representative national sample of 4,668 people, plus data on 2,119 people from two Danish counties collected in the same year. The sampling was based on only two criteria: being a Danish citizen aged 16 years or more and being non-institutionalized. Data were collected through a 45-minute interview and a self-administered questionnaire to be mailed back within two weeks. The inclusion of the additional 2,119 people increased the statistical power to the estimated SF-36 weights. The two counties' samples have the same age and sex pattern as the rest of the surveyed population and do not have any special health characteristics different from the rest of the country.

The specific SF-36 questionnaire was part of the self-administered questionnaire. SF-36 contains 36 items which cover eight dimensions of health-related quality of life: bodily pain (BP), general health (GH), mental health (MH), physical functioning (PF), role emotional limitations due to health (RE), role physical limitations due to health (RP), social functioning (SF), and vitality (VI). Each of the scales gives a score between 0 (worst) and 100 (best). It is possible to calculate the SF-36 scale value for an individual response if at least half of the items in each SF-36 scale are answered. An analysis by Bjørner and colleagues [3] of the same national survey found that the percentage of the Danish population for whom it is possible to calculate the SF-36 dimension scores varies from 93% (general health scale) to 100% (social function scale). The only dimension for which calculating a score was a problem was general health among respondents older than 66 years of age, where it was possible to calculate the scale value for only 79% of the incoming responses. Other studies on the same national sample [4,6] have more details on tests of data quality, scaling assumptions, reliability and validation of the translation of the Danish SF-36.

The 1994 survey also contains a broad range of information about health status, diseases, symptoms, and background information such as age, sex, residence, education, profession, and ethnicity.

Education was defined as a combination of school and vocational training based on the International Standard Classification of Education (ISCED). According to their level of education respondents were placed in three groups: individuals in the low level of education group had less than 10 years of education; those in the middle group had 10–12 years of education; and in the high one individuals had more than 13 years of education.

Income was defined as personal income before tax (DKK/year): low income ranges from 0–99,000; middle income – from 100,000–199,000, and high income – from 200,000 and above.

Information on long-standing diseases originated from an open-ended question "Do you suffer from any long-standing illness, long-standing after-effect from injury, any disability or other long-standing (6+ months) condition?" An affirmative answer led to questions about the specific nature of the illness, including where in the body the illness was located, about how long it had been there, whether a medical doctor had diagnosed it, and whether the illness gave much or little limitation in work and daily life. Answers to questions were coded into 14 main groups according to a modified version of the World Health Organization's *International Classification of Diseases *(ICD-8): infectious/parasitic diseases, malignant neoplasm, diseases of the endocrine system, blood diseases, psychiatric disorders, diseases of the nervous system, cardiovascular diseases, respiratory diseases, digestive diseases, genito-urinary diseases, skin diseases, musculoskeletal diseases, injuries, and other diseases. SF-36 scores were not calculated for disease prevalence lower than 1%.

The survey listed 14 different symptoms and asked the participants if they had experienced these in the two weeks prior to the survey point. The symptoms were: shoulder/neck pain, back pain, arm/leg pain, headache, palpitations, worrying, sleeping problems, melancholy/depression, fatigue, stomach ache, constipation, eczema, cold/rhinitis/coughing, and breathing difficulties.

In the interest of knowing the upper end-point of average SF-36 dimension scores, we defined a subsample of persons who reported having no diseases currently or previously, no long-standing diseases, and no symptoms, pain or complaints.

The statistical methods involved first, applying the original SF-36 scale score algorithm, which from polytomous response categories ranging from 2 to 6 response choices per item, yields a 8-dimensional profile, with each scale having a range from 0 to 100 (100 = optimal) [3,7]. Second, we tabulated the distribution of the eight specific SF-36 dimension scores for respondents reporting different diseases and symptoms, according to sex, income and education, and adjusted for age differences.

The estimation of the relative severity of diseases and symptoms was based on the scores that individuals suffering from the self-reported diseases and symptoms obtained on both the eight single dimensions and on a combined total score, calculated as a simple average of all eight sub-scales.

We used logistic regression analysis to control for the influence of sex, age and SES on the SF-36 combined score after having tested for interaction between these factors and diseases on the SF-36 score. We entered all variables as dummies. The SF-36 combined scale score was dichotomised to over and under 80. The cut-off point at 80 was arbitrarily set on the basis of the distribution of the SF-36 combined scale score for the entire sample (mean females: 80.1, males: 84.7, all: 82.1). From the combined score we ranked the age-adjusted relative severity of diseases separated by sex and educational groups (using the International Standard Classification of Education (ISCED) where low is less than 10 years of education and high is more than 10 years of education).

## Results

The overall response rate to the interviews in the 1994 Danish Health and Morbidity Survey was 79% and to the self-administered SF-36 questionnaire, 64% (N = 5,472). Characteristics of the study population appear in the first column of Table 1. The study includes nearly equal numbers of males (48%) and females (52%). More males and females from the study population are in the higher educational group. More males from the survey are in the highest income group, whereas more females are in the mid income group.

**Table 1:** SF-36 Dimensions and Combined Scores on 8 Dimensions in Age-adjusted Socio-demographic Subgroups and in a Subgroup without Illness (100 = Optimal).

		N	BP	GH	MH	PF	RE	RP	SF	VI	Comb
**Age**	16–24 years	881	83.4	83.2	81.6	96.7	91.7	93.6	95.0	72.9	87.3
	25–44 years	2180	81.5	80.4	82.1	94.6	88.9	90.5	92.8	70.6	85.2
	45–64 years	1691	77.8	73.0	82.1	85.7	87.0	80.8	90.9	70.2	80.9
	65+ years	720	75.8	64.6	80.5	70.6	73.1	59.4	87	65.5	72.1

**Sex**	Males	2828	83.4	77.5	83.9	90.9	89.0	86.5	93.3	72.9	84.7
	Females	2644	76.4	75.4	80.0	86.7	83.6	80.7	90.3	67.6	80.1

**Education**	*Males*										
	Low	613	78.6	70.6	82.3	82.3	82.3	75.7	89.6	69.8	78.9
	Mid	746	82.1	77.0	84.3	89.8	89.4	85.3	93.7	73.0	84.3
	High	1313	86.2	80.2	84.1	94.8	91.0	91.5	94.3	73.9	87.0
	*Females*										
	Low	435	69.4	64.4	77.4	71.8	75.4	64.6	85.9	63.0	71.5
	Mid	864	76.6	75.0	79.8	86.8	85.2	81.0	90.0	67.5	80.2
	High	1234	80.1	79.6	81.4	92.4	88.2	86.7	92.3	69.5	83.8

**Income**	*Males*										
	Low	529	80.8	74.4	81.6	86.2	84.5	79.4	91.0	69.6	80.9
	Mid	759	81.2	75.6	83.2	88.5	86.3	82.8	92.2	72.3	82.7
	High	1294	85.9	80.0	85.2	94.5	92.8	92.2	94.8	74.6	87.5
	*Females*										
	Low	899	72.4	68.3	77.4	78.1	79.2	71.2	87.4	63.9	74.7
	Mid	1262	77.8	77.1	80.7	89.7	86.7	84.1	91.5	68.6	82.0
	High	524	81.0	81.8	82.9	93.4	90.3	88.8	93.1	71.1	85.3

**No illness**	Males	548	95.8	86.7	89.5	95.8	95.7	95.1	97.6	82.3	92.3
	Females	286	94.9	87.0	88.4	95.3	95.4	96.2	97.6	79.6	91.8

**Entire sample**		5472	79.7	76.0	81.8	88.2	86.6	82.9	91.6	70.0	82.1

The nonrespondents have been characterized with respect to gender, age, region, county, and marital status. The odds for nonresponse in the survey increased by age and the increase was gender-specific. For individuals between 15 and 59 years of age, the odds for participation were lower for men than for women. Among people older than 59 years, the odds for non-participation were higher for widowed, divorced, and unmarried people than for married people. Finally, the odds for non-participation increased with urbanization [2,8].

Table 1 shows SF-36 dimension scores and the combined score across the different basic population characteristics of the sample. The age-adjusted SF-36 combined score across the eight dimensions for the entire sample is 82.1, with females and elderly people having lower scores (worse health) than males and younger age groups on all dimensions. The scores on the physical functioning (PF) and role physical (RP) dimensions are lower for the representatives of the 45–64 age group, and together with the scores on the general health (GH) and role emotional (RE) dimensions, they are the lowest for individuals older than 65 years. The respondents with the highest combined scores in the sample, i.e. the healthiest people, are those with no diseases currently or previously, no long-standing diseases, no symptoms, pain or complaints reported. Both males and females reported the highest combined score (97.6) for social functioning (SF). The lowest score in the healthiest group, 79.6, is for females on the vitality index (VI).

Table 2 shows confidence intervals (95%) for various representative sample sizes for each SF-36 dimension score. This table can serve as a tool for the reader when comparing various dimension scores, and is therefore presented here instead of large tables with uncertainties around all estimates.

**Table 2:** 95% Confidence Intervals of SF-36 Dimension Scores According to Different Sample Sizes. 1994 Danish Health and Morbidity Survey (N = 5472).

**SF-36 dimension**	**N = 100**		**N = 500**		**N = 1000**		**N = 2000**		**N = 5472**	
	Lower	Upper	Lower	Upper	Lower	Upper	Lower	Upper	Lower	Upper
Bodily pain	76.5	85.2	77.6	81.8	78.1	81.2	77.9	80.1	79.2	80.4
General health	71.7	80.5	73.2	77.1	74.6	77.4	75.2	77.1	76.0	77.0
Mental health	77.7	84.1	79.7	82.6	80.5	82.6	80.6	82.1	81.4	82.2
Physical functioning	90.8	95.4	87.1	90.8	87.5	90.1	87.8	89.6	88.3	89.3
Role emotional	75.2	89.0	82.6	88.1	83.9	87.7	85.0	87.6	85.9	87.3
Role physical	81.0	92.8	81.1	87.0	80.4	84.6	81.1	84.1	82.8	84.4
Social functioning	91.1	96.9	89.5	92.8	90.4	92.7	90.8	92.4	91.3	92.1
Vitality	64.6	72.9	66.7	70.4	68.0	70.7	68.2	70.1	69.6	70.7

**Figure 1:**
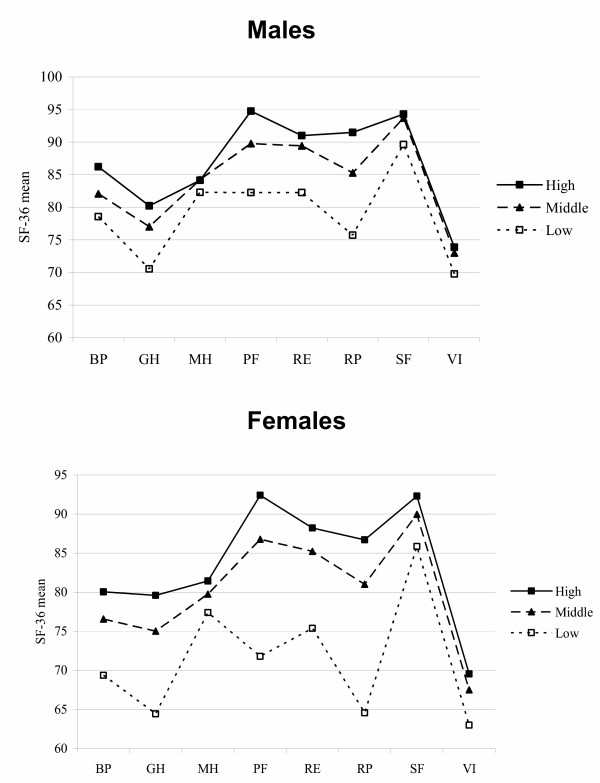
Age-adjusted SF-36 Dimension Scores (100 = Optimal) According to Educational Level (ISCED).

**Figure 2:**
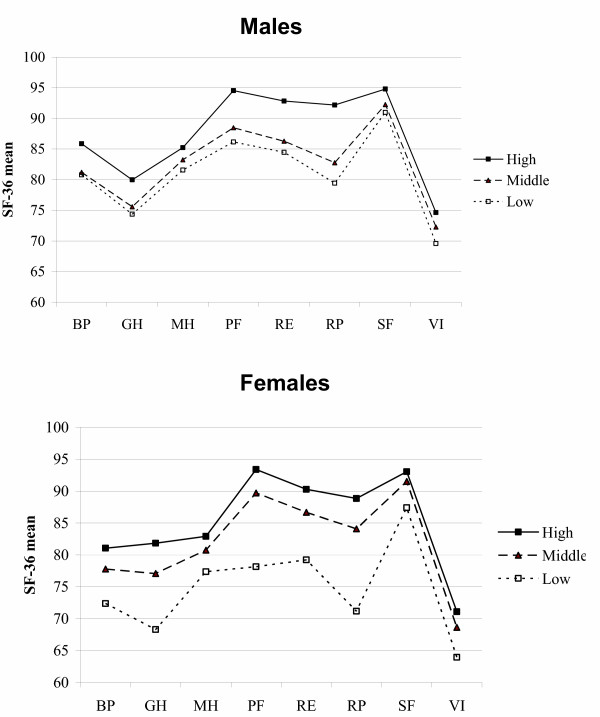
Age-adjusted SF-36 Dimension Scores (100 = Optimal) According to Income Level.

Individuals with lower SES have lower SF-36 scores on all dimensions compared to others. This is seen both with regard to education (Figure 1) and income (Figure 2).

Table 3 shows the total prevalence by sex of 14 different symptoms reported as apparent in the two weeks prior to the survey. Females reported more symptoms than males did for all symptoms in the list. The most commonly reported symptoms for both sexes are pain or discomfort in shoulder and/or neck and back pain. The largest gender difference in symptom prevalence is observed for headache, worrying, depression and general unhappiness, and sleeping problems. All in all, women reported more symptoms than men did, and they also had lower SF-36 scores for all 14 symptoms.

**Table 3:** Prevalence of Symptoms and Ranking of Symptoms According to Age-adjusted SF-36 Dimensions and Combined Scores (100 = Optimal) for Males and Females.

**Sex**	**Rank**	**Symptom**	**Prevalence %**	**BP**	**GH**	**MH**	**PF**	**RE**	**RP**	**SF**	**VI**	**SF-36 comb**
**Males**	1	Melancholy, depression	3.1	70.7	59.2	57.9	83.3	56.3	70.0	71.4	51.2	65.0
	2	Breathing difficulties	4.1	62.8	52.2	75.5	72.3	68.6	64.8	82.1	53.5	66.5
	3	Worrying	2.9	68.9	59.3	61.9	87.2	59.7	73.7	74.0	52.0	67.1
	4	Palpitations	2.6	70.3	62.8	71.6	82.6	70.4	74.2	80.0	57.9	71.2
	5	Sleeping problems	5.4	71.0	65.0	71.5	83.1	74.2	71.8	82.0	57.0	72.0
	6	Stomach ache	3.8	68.1	66.4	75.9	86.0	78.1	72.4	84.3	61.4	74.1
	7	Constipation	2.9	74.9	65.9	76.5	86.0	76.4	75.7	85.1	61.0	75.2
	8	Fatigue	9.8	76.3	66.5	75.9	87.4	82.7	76.2	86.3	57.4	76.1
	9	Eczema	4.1	77.5	69.2	77.6	86.6	79.0	78.3	88.3	64.7	77.7
	10	Arm, leg pain	14.4	69.0	71.2	81.8	85.9	84.5	77.1	90.6	66.8	78.4
	11	Back pain	18.5	69.9	71.6	80.9	87.3	85.7	79.2	90.8	66.2	78.9
	12	Headache	11.1	73.5	71.9	80.1	90.1	85.5	81.3	88.9	65.4	79.6
	13	Shoulder, neck pain	17.3	74.0	74.2	81.0	90.3	85.7	82.6	91.7	67.6	80.9
	14	Colds, rhinitis, coughing	11.3	82.9	74.7	82.1	89.9	86.2	82.2	91.0	69.0	82.3
**Females**	1	Melancholy, depression	7.4	63.7	60.4	57.4	76.0	56.4	61.7	74.1	49.0	62.3
	2	Breathing difficulties	4.7	62.5	55.2	72.5	68.9	64.5	57.1	80.9	53.6	64.4
	3	Worrying	6.0	66.8	62.1	62.3	78.2	60.5	64.4	77.3	52.8	65.5
	4	Fatigue	14.2	61.7	59.9	70.4	76.7	71.0	58.6	79.9	49.3	65.9
	5	Palpitations	4.2	61.8	61.6	68.4	74.7	67.6	60.7	82.1	54.9	66.5
	6	Sleeping problems	10.9	62.8	62.1	67.8	77.2	66.0	62.8	80.0	53.3	66.5
	7	Stomach ache	6.8	57.8	62.4	71.2	77.6	74.6	65.7	79.9	54.9	68.0
	8	Constipation	7.0	63.3	64.2	71.5	78.4	71.9	63.9	81.6	55.2	68.8
	9	Back pain	25.0	61.4	65.0	75.0	78.5	75.7	65.6	84.5	57.7	70.4
	10	Arm, leg pain	21.2	61.4	65.0	75.0	78.5	75.7	65.6	84.5	57.7	70.4
	11	Headache	19.0	65.3	68.2	74.4	81.2	81.0	69.9	84.1	59.9	73.0
	12	Shoulder, neck pain	29.3	65.9	68.6	75.9	81.0	79.6	71.6	86.2	60.6	73.7
	13	Eczema	6.4	69.6	65.5	78.1	80.0	84.2	69.6	85.7	59.5	74.0
	14	Colds, rhinitis, coughing	12.0	74.4	70.3	78.2	84.7	81.9	74.6	87.7	64.9	77.1

For both sexes, the two most severe reported symptoms according to age-adjusted SF-36 combined score are melancholy/depression (males: 65.0, females: 62.3) and breathing difficulties (males: 66.5, females: 64.4). For the commonly reported symptoms back pain and shoulder/neck pain, the average rankings are at numbers 11 and 13 respectively for males and at 9 and 12 for females. Younger people reported primarily the mildest symptoms: headache, shoulder/neck pain, colds/rhinitis/coughing, and fatigue (not in Table). Elderly people reported greater health problems such as breathing difficulties, palpitations, constipation, sleeping problems, and pain in arms/legs (not in Table). The relationship between prevalence and consequences of symptoms seems in general to be inverse, i.e. the more prevalent the symptom, the less severe the reported consequences.

**Figure 3:**
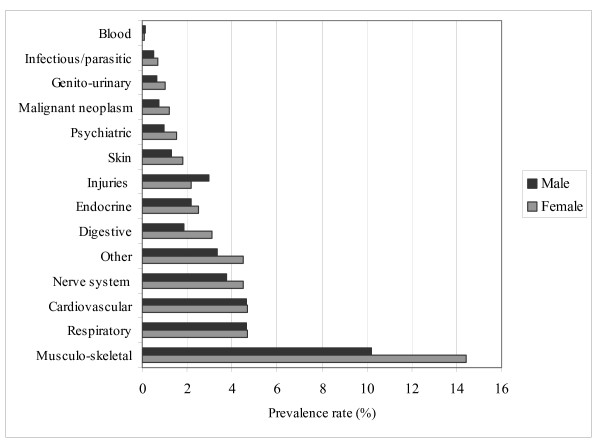
Prevalence of Self-reported Diseases for Males and Females.

Figure 3 shows the self-reported diseases by sex. The prevalence is highest for musculoskeletal diseases (males: 10.2%, females: 14.4%), cardiovascular diseases (males: 4.6%, females: 4.7%), and respiratory diseases (males: 4.6%, females: 4.7%). With the exception of injuries and blood diseases, females reported more of all diseases than males did.

Table 4 presents the results of the logistic regression analysis relating sex, age, education, and diseases to the SF-36 combined scores. Model 1 shows that sex, age, and education are significantly related to the SF-36 combined score. Higher educational groups have a higher SF-36 combined score. Model 2 demonstrates a statistically significant relationship between all diseases, except skin diseases, to lower SF-36 scores, with the largest effect for psychiatric disorders. On the SF-36 combined score, we found no interaction between sex and diseases, while there was interaction between age and respiratory diseases and skin diseases. Model 3 estimates the full model containing both education and diseases and the interaction between them on the SF-36 combined score. We observed interaction between education and both respiratory and musculoskeletal diseases in relation to the SF-36 combined score. Only these two diseases are shown in model 3. No interaction was established between income levels and any of the investigated disease groups in relation to SF-36 (not in Table).

**Table 4:** The Influence of Sex, Age, Education and Diseases on SF-36 Combined Score by Logistic Regression Analyses.

	**Model 1**			**Model 2**			**Model 3**		
Explanatory variables	Socio-demographic group			Disease group			Socio-economy and diseases		
			
	**B**	**S.E.**	**Sig.**	**B**	**S.E.**	**Sig.**	**B**	**S.E.**	**Sig.**
Constant	-0.675	0.103	**0.000**	0.521	0.107	**0.000**	0.377	0.136	**0.005**
Sex	0.511	0.069	**0.000**	0.549	0.073	**0.000**	0.501	0.075	**0.000**
*Age (years)*									
16–24	1.518	0.141	**0.000**	1.187	0.139	**0.000**	1.081	0.155	**0.000**
25–44	1.174	0.104	**0.000**	0.932	0.113	**0.000**	0.829	0.118	**0.000**
45–64	0.805	0.103	**0.000**	0.712	0.113	**0.000**	0.664	0.116	**0.000**
*Socio-economic group*									
Education	0.568	0.083	**0.000**				0.306	0.120	**0.011**
*Disease*									
Cardiovascular				-1.245	0.160	**0.000**	-1.107	0.286	**0.000**
Malignant neoplasm				-0.854	0.304	**0.005**	-2.484	1.094	**0.023**
Endocrine diseases				-0.819	0.211	**0.000**	-1.016	0.442	**0.022**
Psychiatric disorders				-2.370	0.331	**0.000**	-2.486	0.791	**0.002**
Nervous system				-0.805	0.158	**0.000**	-0.471	0.314	0.133
Respiratory diseases				-0.948	0.147	**0.000**	-1.842	0.358	**0.000**
Digestive diseases				-1.051	0.218	**0.000**	-0.959	0.414	**0.020**
Genito-Urinary diseases				-0.749	0.368	**0.042**	-0.905	0.927	0.329
Skin diseases				-0.454	0.244	0.063	-0.380	0.574	0.508
Muscular-skeletal diseases				-1.469	0.094	**0.000**	-1.817	0.201	**0.000**
Injuries				-0.607	0.191	**0.002**	0.045	0.461	0.922
Other diseases				-0.999	0.162	**0.000**	-1.832	0.450	**0.000**
*Interaction:*									
ISCED * Respiratory							1.219	0.400	**0.002**
ISCED * Muscular-skeletal							0.485	0.230	**0.035**

Coding

Sex: female = 0, male = 1

Age: baseline category: 65+

ISCED: low = 0, high = 1

SF-36 comb: score 0–79 = 0, score 80–100 = 1

Tables 5 and 6 show the prevalence and severity of diseases for males and females. Due to the interaction between education and some diseases on the SF-36 combined score, which is found in the logistic regression, the tables are stratified by education. Males with relatively low education reported more of nearly all diseases than males with higher education did. 25.8% of females with less education and 14.1% of females with higher education claimed that they suffered from musculoskeletal diseases. At the same time, 21.2% of males with less education and 11.8% with higher education reported suffering from these diseases. Cardiovascular diseases were the second most reported disease group for both sexes, but with a threefold difference in frequency between low educated females and those with higher education (low: 12.6%, high: 3.6%). Respiratory diseases were the third most reported disease group, also more frequently reported by individuals with low education. Females reported more of all diseases than males did, except for injuries among respondents with higher education.

**Table 5:** Prevalence and Ranking of Disease Groups According to Age-adjusted SF-36 Dimensions and Combined Scores (100 = Optimal) on Educational Groups (ISCED). Males.

**ISCED**	**Rank**	**Disease**	**Prevalence %**	**BP**	**GH**	**MH**	**PF**	**RE**	**RP**	**SF**	**VI**	**SF-36 Comb**
**High**	1	Psychiatric disorders	1.4	74.9	56.0	68.0	87.7	54.4	63.3	72.3	56.4	66.6
	2	Respiratory diseases	5.0	79.5	66.3	81.0	84.1	57.6	76.5	91.2	67.1	75.4
	3	Cardiovascular diseases	5.2	80.0	64.4	79.1	86.5	71.7	71.3	86.5	66.3	75.7
	4	Injuries	3.7	68.5	73.4	80.5	81.5	86.1	77.6	89.1	67.7	78.1
	5	Nervous system	4.4	77.6	70.2	80.1	86.0	78.7	76.3	90.0	69.1	78.5
	6	Musculoskeletal diseases	11.8	69.6	71.1	81.7	85.7	82.4	78.8	92.0	68.0	78.7
	7	Skin diseases	1.3	69.6	71.1	81.7	85.7	84.6	78.8	92.0	68.0	78.9
	8	Digestive diseases	2.0	73.1	74.9	80.6	89.3	81.1	82.1	94.5	64.6	80.0
	9	Other diseases	3.5	80.0	68.6	83.9	89.4	81.5	81.6	92.4	69.0	80.8
	10	Endocrine diseases	2.3	86.3	66.8	79.7	91.0	79.4	88.6	93.2	68.3	81.7
**Low**	1	Psychiatric disorders	1.4	48.2	20.6	53.2	67.5	54.4	58.0	62.4	36.6	50.1
	2	Respiratory diseases	6.1	58.5	43.0	70.7	61.0	57.6	43.7	80.0	49.5	58.0
	3	Endocrine diseases	4.2	55.4	47.5	79.8	59.4	79.4	29.7	83.4	56.1	61.3
	4	Other diseases	5.0	59.6	52.0	67.9	62.7	81.5	68.7	65.3	50.8	63.6
	5	Digestive diseases	4.5	58.7	53.7	80.0	62.9	81.1	53.0	81.8	58.8	66.3
	6	Skin diseases	2.2	62.5	50.3	81.5	65.9	84.6	61.9	80.4	56.2	67.9
	7	Cardiovascular diseases	9.3	67.9	52.2	77.4	69.5	71.7	64.2	80.6	62.3	68.2
	8	Musculoskeletal diseases	21.2	59.1	58.6	78.9	72.1	82.4	59.2	85.2	59.5	69.4
	9	Nervous system	5.8	77.3	67.2	78.3	81.8	78.7	68.6	84.3	68.0	75.5
	10	Injuries	3.7	68.6	69.3	89.2	78.7	86.1	72.6	95.5	79.2	79.9

**Table 6:** Prevalence and Ranking of Disease Groups According to Age-adjusted SF-36 Dimensions and Combined Scores (100 = Optimal) on Educational Groups (ISCED). Females.

**ISCED**	**Rank**	**Disease group**	**Prevalence %**	**BP**	**GH**	**MH**	**PF**	**RE**	**RP**	**SF**	**VI**	**SF-36 comb**
**High**	1	Psychiatric disorders	1.5	69.3	53.7	64.1	77.7	54.4	59.2	72.7	51.3	62.8
	2	Digestive diseases	2.8	57.4	63.4	70.7	81.0	81.1	71.0	81.1	53.2	69.9
	3	Cardiovascular diseases	3.6	67.1	59.9	75.6	79.3	71.7	63.6	84.2	58.3	70.0
	4	Musculoskeletal diseases	14.1	57.9	64.3	75.9	76.5	82.4	64.5	85.8	59.8	70.9
	5	Respiratory diseases	4.9	70.9	64.8	80.2	78.9	57.6	74.5	87.0	62.4	72.0
	6	Other diseases	5.1	66.5	65.9	77.1	80.9	81.5	67.6	84.9	60.4	73.1
	7	Nervous system	4.4	67.7	64.2	77.7	81.4	78.7	71.2	86.5	62.1	73.7
	8	Injuries	2.4	67.6	74.9	79.6	83.2	86.1	74.5	86.6	67.0	77.5
	9	Skin diseases	2.2	75.2	67.2	76.2	83.1	84.6	79.4	91.7	63.3	77.6
	10	Endocrine diseases	2.2	78.4	69.8	80.4	86.5	79.4	81.0	91.2	67.6	79.3
**Low**	1	Respiratory diseases	7.2	59.1	43.4	72.4	58.3	57.6	35.4	84.4	47.9	57.3
	2	Psychiatric disorders	3.2	52.3	54.9	61.9	70.9	54.4	48.1	74.3	54.9	58.9
	3	Other diseases	4.9	54.4	51.0	66.8	55.3	81.5	47.9	76.0	48.5	60.2
	4	Cardiovascular diseases	12.6	58.3	51.1	71.2	56.7	71.7	49.5	77.8	50.2	60.8
	5	Musculoskeletal diseases	25.8	50.8	51.3	74.5	57.5	82.4	44.5	77.7	53.5	61.5
	6	Nervous system	9.2	55.0	54.5	74.9	62.7	78.7	47.8	76.3	57.1	63.4
	7	Endocrine diseases	6.4	66.8	58.0	76.5	61.5	79.4	48.1	81.8	58.8	66.4
	8	Digestive diseases	6.3	58.6	56.0	77.2	64.5	81.1	55.0	81.0	60.2	66.7
	9	Skin diseases	1.9	71.4	67.8	85.5	82.7	84.6	74.6	93.1	67.6	78.4
	10	Injuries	3.8	69.7	69.0	85.5	85.9	86.1	81.0	91.0	66.3	79.3

As for the ranking of the SF-36 combined score of all dimensions, psychiatric disorders ranked number one for both males and females with higher education (Tables 5 and 6). Among individuals with lower education, the most severe reported conditions were respiratory diseases for females and psychiatric disorders for males. The overall combined score for psychiatric disorders was lower for males (worse health) than for females. The score for males with low education on the SF-36 combined score was extremely low (50.1), and it was even lower on the general health dimension (20.6) and the vitality index (36.6). For all other reported diseases female scores were substantially lower than male scores. The most frequently reported disease group, musculoskeletal diseases, was ranked with a higher relative severity by males and females with high levels of education. Females with low education had very low scores on the role physical (44.5) and bodily pain (50.8) dimensions.

## Discussion

We have used the original eight dimension scales in the SF-36, as well as an average of all dimensions combined, to rank diseases and symptoms. A recent study [9] measured the relative severity of seven chronic diseases in eight countries (Denmark, France, Germany, Italy, Japan, the Netherlands, Norway, and USA). By using multivariate linear regression on the two standard SF-36 summary measures for mental health (consisting of VT, SF, RE, MH dimension scales) and physical health (PF, RP, BP, GH dimension scales), it was found that hypertension, allergies, and arthritis were the most frequently reported conditions, while arthritis, chronic lung disease, and congestive heart failure were the most severe ones. Also Sprangers and colleagues [10] used the standard SF-36 summary measures for mental and physical health to estimate the relative severity of chronic diseases. They established that patients who were older, female, with low education, who lived alone and had at least one co-morbid condition, in general reported the poorest level of quality of life as measured by the SF-36. Differences in background variables and disease groups make comparability across populations difficult, even with a standardized questionnaire like the SF-36. In addition, there is a likely difference in reporting behaviour and health norms across populations and groups. However, the results seem in general to be in concordance with our findings.

Considerable evidence suggests response shifts from self-reported health data both across countries and within countries across age, sex, and socio-economic groups [11]. Different new strategies have been identified for adjusting for known biases and enhancing the cross-population comparability [12], which would be attractive to apply to SF-36 self-reported data in future studies.

In our study, besides looking at long-standing diseases, we included a list of symptoms. We found that the most frequently reported symptoms were shoulder, neck, back, arm, leg problems, and headache. These symptoms are probably mostly related to musculoskeletal problems. When comparing musculoskeletal diseases with reported shoulder, neck and back problems, diseases proved to be more severe than symptoms, which was reflected in lower SF-36 combined scores. Psychiatric disorders were ranked relatively high with regards to severity of diseases but had a relatively low self-reported frequency (1–1.5%). On the other hand, melancholy/depression, the most severe symptom for both sexes, had a moderately high frequency of reporting (3–7%). It is important to be aware of these relationships for the planning of resource allocation in preventive, curative, and rehabilitative health services.

Since consequences here are measured on a continuous scale, from 0 to 100 (from worst health or most consequences to optimal health or fewest consequences), they would be relatively easy to use as necessary inputs in constructing summary measures of population health, like QALY, DALY, and HALE. A comparison of the self-reported SF-36 combined raw scores from the Danish survey (not stratified by sex, age, and SES) and the hypothetical expert-evaluated disability weights from 15 roughly comparable single conditions from the Global Burden of Disease Study [13] and from the Dutch Disability Weight Project [14] showed high Pearson correlation coefficients of 0.742 (p < 0.01) and 0.850 (p < 0.01) respectively.

Therefore, not only in our study the SF-36 indicates relatively high average scores on all scales. Even among persons reporting diseases, the average scale scores are usually high (50+), and for self-reported completely healthy persons we can rarely obtain over 90 in average scale scores. This is possibly due to a problem with the SF-36 scale's arbitrary scoring system.

We have observed large gender differences in our study according to severities of symptoms and diseases. Females reported more frequently and with higher severities all symptoms and most diseases than males did. This is an important finding because it shows that the higher self-reported female morbidity is not caused by an over-reporting of milder causes only. It is likely, but only speculative based on our data, that it reflects the existence of real gender differences in health, not only a gender gap resulting from different perceptions or willingness to report illness. Research in gender differences in health has pointed towards the different social roles. For example, MacIntyre et al. [15] claim that the many societal changes during the last decades are likely to have produced differences in male and female health status. However, this appears to be not entirely correct considering for example Lindhardt's [16] investigation from Denmark in the 1950s, which showed almost the same gender gap in patterns of morbidity as we see today.

Socio-economic differences in the SF-36 combined scores were apparent when both income and education were used. The SF-36 combined score was much lower for less educated and low-income groups than for others with higher education and income, with the exception of the mental health domain. The SES gradient is also found for the same survey population in relation to self-reported long-standing illness and perceived general health [17]. Our results show that a low educational level is associated with higher disease prevalence compared to higher educational levels, especially for reported cardiovascular, musculoskeletal and endocrine diseases, and for females, psychiatric disorders and respiratory diseases as well. Based on the logistic regression analysis we determined that reported respiratory and musculoskeletal diseases have interaction with education on the SF-36 combined score. This means that the SF-36 combined score for a given self-reported disease is dependent on the educational class to which people belong. This affects the ranking of diseases, so ranking should be separate for different educational groups. Burström and colleagues [18] utilized the five domains of EuroQol to obtain mean health-related quality of life weights from a survey of the Swedish population. They found a gradient of health-related quality of life by age, sex, and SES that is consistent with our results from Denmark using the SF-36. For most diseases females reported more problems in each of the domains of EuroQol, with the exception of self-care. Males with depression and asthma reported more problems in the EuroQol anxiety/depression domain than females did. Interaction between SES and diseases was not found in the study.

Interpretations of the results from studies that aim to measure health-related quality of life in specific patient groups are very dependent on the availability of similar measurements from reference groups, the so-called "normal" populations. It is important to know the occurrence of the different measures of quality of life in a normal population, as well as factors other than illness itself, e.g. treatment of diseases, that potentially influence the study population's quality of life. The present study could be used as a reference study of the Danish adult population.

Construct validity and differential item functioning in the SF-36 from the same Danish survey have been thoroughly analysed in other studies [3-6]. The variation by age and sex showed that on all SF-36 dimensions males scored higher than females (better health). For dimensions that reflect physical health there was a tendency to lower scores by age (worse health). For scales reflecting mental health, there was no clear age pattern. The inventors of the SF-36 point out that the instrument is suitable for self-administration and has been administered successfully in general population surveys as well as to young and old adult patients with specific diseases [19]. However, a British study found systematic differences in health ratings for the SF-36 by mode of administration. Postal self-completed response ratings were systematically lower than interview-administrated ratings [20]. Some studies also express concerns about using the questionnaire for older people [21,22].

We could have chosen not to adjust self-reported diseases and symptoms for age differences, since it would standardize differences in severity that obviously are influenced by age. If we were interested in having a measure for the actual reported consequences of the diseases and symptoms from a need-based care perspective, it would be most logical to use the un-standardized scores. Older people would naturally have the highest disease burden because they suffer from more symptoms and diseases with age. However, since we are interested in being able to use results for international comparative studies of the individual burden with focus on the severity of diseases and symptoms regardless of individual age, we have used indirect age standardization.

In conclusion, using a combined score aggregated on the basis of the eight SF-36 dimensions seems to be a feasible way to rank diseases and symptoms to assess empirical population reporting of health status. Musculoskeletal symptoms and diseases are the most commonly reported, while psychiatric disorders and respiratory diseases are the most severe diseases reported. Sex, age, and SES affect the SF-36 score. However, certain reported diseases also interact with SES (education), which prevents estimating the severity as an unambiguous disease-specific SF-36 combined score. The difference in severity according to SES means that a single reported disease will appear more or less severe, i.e. there will be a change in its rank ordering depending on the SES. Our recommendation is, therefore, that severity estimates for diseases and symptoms be interpreted with caution or stratified for socio-economic groups.
